# Effects of pre- and postnatal nutrition interventions on child growth and body composition: the MINIMat trial in rural Bangladesh

**DOI:** 10.3402/gha.v6i0.22476

**Published:** 2013-12-13

**Authors:** Ashraful Islam Khan

**Affiliations:** 1 icddr,b, Dhaka, Bangladesh; 2 Department of Women’s and Children’s Health, International Maternal and Child Health, Uppsala University, Uppsala, Sweden

**Keywords:** body composition, child growth, food supplementation, multiple micronutrients, pregnancy

## Abstract

**Background:**

Nutritional insults and conditions during fetal life and infancy influence subsequent growth and body composition of children.

**Objectives:**

Effects of maternal food and micronutrient supplementation and exclusive breastfeeding counseling on growth of offspring aged 0–54 months and their body composition at 54 months of age were studied.

**Methods:**

In the MINIMat trial (ISRCTN16581394) in Matlab, Bangladesh, pregnant women were randomized to early (around 9 weeks) or usual invitation (around 20 weeks) to food supplementation and to one of the three daily micronutrient supplements: 30-mg Fe and 400-µg folic acid (Fe30F), 60-mg Fe and 400-µg folic acid (Fe60F), and multiple micronutrient supplements (MMS). The supplements were also randomized to exclusive breastfeeding (EBF) counseling or to usual health messages.

**Results:**

No differences in background characteristics were observed among the intervention groups. There was also no differential effect of prenatal interventions on birthweight or birthlength. Early food supplementation reduced the level of stunting from early infancy up to 54 months of age among boys (average difference – 6.5% units, 95% confidence interval [CI] 1.7–11.3, *p*=0.01) but not among girls (average difference – 2.4% units, 95% CI −2.2–7.0, *p*=0.31). MMS resulted in more stunting compared to standard Fe60F (average difference – 4.8% units, 95% CI 0.8–8.9, *p*=0.02). Breastfeeding counseling prolonged the duration of EBF (difference – 35 days, 95% CI 30.6–39.5, *p*<0.001). Neither pregnancy interventions nor breastfeeding counseling influenced the body composition of children at 54 months of age.

**Conclusion:**

Early food supplementation during pregnancy reduced the occurrence of stunting among boys aged 0–54 months, while prenatal MMS increased the proportion of stunting. Food and micronutrient supplementation or EBF intervention did not affect body composition of offspring at 54 months of age. The effects of prenatal interventions on postnatal growth suggest programming effects in early fetal life.

The relevance of nutrition during pregnancy and in early infancy and its long-term effect on subsequent health of the offspring is highlighted in the Developmental Origins of Health and Disease (DOHaD) hypothesis (1–3). Early-life conditions influence growth patterns, body composition, and subsequent risk for non-communicable chronic diseases (4). Prenatal food supplementation has been used as one of the strategies to improve maternal nutritional status and fetal development where food insecurity and maternal malnutrition are prevalent (5–9). These interventions have shown mixed results (5–12) due to variations in maternal nutrition status, amount and composition of food supplements, seasonal differences in food availability, and also some other factors that may influence maternal nutritional status and fetal growth (13). However, optimal timing of food supplementation to malnourished pregnant women is unclear. Multiple micronutrient supplements (MMS) during pregnancy is one of the strategies to prevent adverse pregnancy outcomes (14, 15). MMS were, therefore, developed for trial (16). Over the past decade, a series of randomized controlled trials that compared MMS with usual iron–folic acid supplement was carried out in Bangladesh (17), China (18), Indonesia (19, 20), Nepal (21), Pakistan (22), Burkina Faso (23), Guinea-Bissau (24), and Niger (25). Meta-analysis of these trials suggests little positive effect of MMS on birthweight (pooled estimate: +22.4 g; 95% confidence interval [CI] 8.3–36.4 g) and a reduction in the prevalence of low-birthweight (LBW) (pooled odds ratio [OR]: 0.89; 95% CI 0.81–0.97) (26).

Knowledge about possible combined effect of prenatal food and MMS on offspring outcomes, including postnatal growth, is lacking. A recent randomized trial in Burkina Faso showed that prenatal multiple micronutrient-fortified food supplements resulted in higher birthlength compared to a single MMS alone (27). As women in disadvantaged settings may suffer from both macro- and micronutrient deficiencies, it is important to investigate whether a combination of nutritional interventions during pregnancy is needed for favorable short- and long-term outcomes in the offspring.

Long-term effects of breastfeeding are more difficult to assess; however, epidemiological data suggest that breastfeeding is associated with a reduced risk of obesity and related metabolic disorders in the later life (28). Some studies, however, could not demonstrate any association between breastfeeding and anthropometric or body-composition indices (29, 30). In contrast, two randomized trials of infant formula of different compositions demonstrated that a nutrient-enriched diet in infancy increases fat mass (FM) later in childhood, supporting a causal link between infant feeding and a later risk of obesity (31). Thus, results of observational studies mainly suggest that breastfeeding has beneficial effects beyond infancy.

## Rationale for the study

The major part of evidence for the DOHaD hypothesis was derived from studies, where birthweight was used as a proxy measure for nutritional state in pregnancy. Epidemiological studies usually investigating an association between prenatal influences and adult chronic diseases and retrospective cohorts are performed in high-income settings that are register-based. However, the DOHaD hypothesis is of particular relevance to low-income settings where poor nutrition in early life is common and, at the same time, a rapid nutrition transition is ongoing and chronic diseases increasingly occur in adult life. Although several prenatal nutritional supplementation trials have been carried out, few have ever investigated effects beyond the neonatal period. Despite the animal experimental evidence and observations from famines, no trials investigated effects of timing of prenatal nutrition intervention. Besides, no trials investigated effects of timing of food supplementation combined with different micronutrient alternatives on outcomes of the offspring, including postnatal growth. The Maternal and Infant Nutrition Intervention in Matlab (MINIMat) trial where random allocation of time at the start of prenatal food supplementation was combined with different micronutrient alternatives is a unique resource for improved knowledge about programming of later health and disease risk (12, 32). MINIMat women were also randomized to exclusive breastfeeding counseling (EBC) or usual health messages (UHM) to see the effect on child growth (33). A separate methodological paper was also published (34). This area of research is particularly important for countries, such as Bangladesh, where the burden of both fetal growth restriction and mortality due to infectious diseases is still heavy and where there is a rapid emergence of overweight, insulin resistance, and chronic diseases. The offspring of this study have been extensively followed up and the current thesis is based on detailed anthropometric measurements from birth to 54 months and body-composition assessment at 54 months of age.

## Aim

Based on the MINIMat trial in rural Bangladesh, the present study was conducted to assess the effects of timing of prenatal food supplementation and different alternatives of micronutrient supplementation and of an exclusive breastfeeding (EBF) counseling intervention on growth and body composition of the offspring.

## Methods

### Study site

The MINIMat (ISRCTN16581394) was conducted in Matlab, a rural subdistrict situated near a river delta, prone to frequent flooding. Matlab is located 57 km southeast of the capital Dhaka in Bangladesh. In Matlab, the icddr,b (International Centre for Diarrhoeal Disease Research, Bangladesh) runs a health and demographic surveillance system (HDSS), which records health and demographic information on a monthly basis since 1966. It also runs a central hospital and four connected subcenters that provide healthcare to the resident population in the areas. The icddr,b has divided the Matlab HDSS into two areas: the Maternal and Child Health and Family Planning Programme (MCH-FP) area (70 villages) and a government service area (79 villages). In the MCH-FP area, female community health research workers (CHRW) visit each household every month and provide intensive healthcare services. In the government service area, the Government provides family planning and basic healthcare services.

### Study design and participants

All pregnant women of the icddr,b service area were eligible for enrolment into the study. The CHRWs visited households to ask women about their menses. Women who reported missing a menstrual period for 2 weeks or more were offered a pregnancy test, and if positive and fulfilling the eligibility criteria (fetus was viable, gestational age <14 weeks by ultrasound examination; the woman had no severe illness, and consented for participation), they were invited to participate in the MINIMat trial.

The primary outcomes of the MINIMat trial were to analyze the effects of prenatal food and micronutrient supplementation on birthweight, infant mortality, and maternal hemoglobin in the third trimester. Several secondary outcomes were defined, including infant and child growth, morbidity, micronutrient status, and cognitive development. This article is based on exposure data from pregnancy (prenatal intervention) and anthropometric outcome data (weight, height) from birth to 54 months of age, and body-composition (fat and fat-free mass [FM and FFM]) data at 54 months of age.

Data were collected during pregnancy and follow-up while making scheduled visits to home and subcenter clinics. During November 2001–October 2003, 4,436 pregnant women were enrolled into the MINIMat study. On enrolment, weights and heights of the women were measured. Trained data collectors interviewed them about their age, parity, level of education, employment, and other socioeconomic information. During February 2007–March 2009, 2,735 children aged 54 months were tracked and followed up. Data were collected at local subcenters run by the icddr,b in the Matlab area, and a health worker from the study accompanied the participants to the study location early in the morning, following an overnight fasting. One of the two teams conducted all measurements. Each team consisted of a medical doctor, a nurse, and a laboratory technician, assisted by the trained field staff.

A methodological study was carried out to assess accuracy of the Tanita TBF 300A leg-to-leg bioimpedance analyzer among Bangladeshi children and to develop a novel equation for the estimation of FFM (35) using deuterium dilution technique as the reference method among a subsample of 200 children (102 males and 98 females) aged 4–10 years.

### Interventions

Pregnant women enrolled into the MINIMat trial were randomly assigned to two food supplementation groups (early invitation: immediately after identification of pregnancy, usually around week 9 or usual invitation: at the time of their choosing, i.e. usual care in this community, in this trial around week 20). An energy–protein food supplement was made available from the ongoing government-supported national program for pregnant women with a body mass index (BMI) of <18.5 kg/m^2^ at the time of data-collection. In the MINIMat trial, this food supplement was offered to all the pregnant women, irrespective of their nutritional status assessed by BMI. The food supplement was locally produced and made available through the community nutrition centers (CNCs) for 6 days a week. The supplement was to be mixed with water containing 80-g roasted rice powder, 40-g roasted pulse powder, 20-g molasses, and 12-mL (6 g) soybean oil, which provided 608 kcal (2.85 mJ). Food was supplemented until the end of pregnancy.

The enrolled pregnant women were also randomly assigned at the clinic visit at 14 weeks of gestation to one of the three micronutrient groups: Capsules containing (1) 30-mg Fe fumarate + 400-µg folate (Fe30F), (2) 60-mg Fe (fumarate) + 400-µg folate (Fe60F), or (3) MMS (15 micronutrients including 30-mg Fe and 400-µg folic acid). The three types of micronutrient supplements taken daily looked identical and were distributed in special pill bottles. The interviewers provided each bottle containing 35 tablets to enrolled pregnant women at home during their monthly home visits, and the supplementation continued up to 3 months postpartum.

All enrolled pregnant women with viable fetuses were individually randomized around 30 weeks of gestational age to receive either EBF counseling (EBC) by the trained counselors or to receive standard/usual health messages (UHM) delivered by the government or icddr,b health staff. Women of the breastfeeding counseling intervention group received counseling in eight sessions: two sessions during the last trimester of pregnancy, one session within 7 days after delivery, and five sessions at monthly intervals up to 6 months after delivery. The importance of EBF for up to 6 months of life was emphasized during counseling. Women of the UHM group received basic and usual health messages about breastfeeding practices during visits to the postnatal clinic with less individualized support. The messages included: benefits of giving colostrum immediately after birth, advice on EBF for 6 months, and advice to start complementary feeding from 6 months along with continuation of breastfeeding until 2 years. The EBC group received UHM plus additional counseling inputs given by the counselors.

### Measurements

Anthropometry at birth was part of the primary outcomes of this trial. Weight and length/height were thereafter measured every month up to 1 year, thereafter every 3 months up to 24 months, and again at 54 months of age. Birth anthropometry was usually performed within 72 hours after birth. Birthweights were measured using the SECA electronic or beam scales (UNICEF Uniscale; SECA Gmbh & Co, Hamburg, Germany), with a precision of 0.01 kg. A locally manufactured, collapsible length board, with a precision of 0.1 cm, was used for measuring recumbent length of the newborn. Maternal weight and height were measured on enrolment at around 8 weeks of gestation. Maternal weight was measured using an electronic scale (Uniscale; SECA), with a precision of 0.10 kg, and height was measured to the nearest 0.1 cm using a stadiometer.

At 54 months of follow-up, bodyweight was recorded to the nearest 0.1 kg using a digital scale (TANITA HD–318, Tanita Corporation, Japan) in light clothing and bare foot. After removing shoes, height was measured to the nearest 0.1 cm using a daily-calibrated freestanding stadiometer Leicester Height Measure (Seca 214, UK). Weighing equipment was calibrated daily with standard weights. Measurements of weight and length/height were converted to weight-for-age, length/height-for-age, and weight-for-length/height z-scores (SD scores) according to the child growth standards of the WHO Multicentre Growth Reference Study (36). Mid-upper-arm circumference (MUAC) was measured to the nearest 0.1 cm with a nonelastic metric-measuring tape at the midpoint of the upper arm, with the arm hanging straight by the subject’s side. Skinfold thickness was measured in triplicate to the nearest 0.2 mm at four sites (biceps, triceps, subscapular, and suprailiac) using the Holtain calipers (Holtain, Crymych, UK). All skinfold measurements and MUAC were also performed in the same order on the left side of the body.

### Bioelectrical impedance analysis

Body composition was assessed by leg-to-leg bioelectrical impedance analysis (BIA) using the Tanita TBF-300MA Body Composition Analyzer (Tanita Corporation, Tokyo, Japan). BIA measurements were made following the guidelines of the manufacturer and at a measurement frequency of 50 kHz. Data relating to height, sex, and age were entered manually while weight was recorded automatically using 0.5 kg as an adjustment of weight for clothes in all subjects. The Tanita software uses inbuilt equations to estimate fat and FFM. These inbuilt equations are based on the Caucasian populations aged 7 years and older, and their validity may be questioned when applied to other ethnic groups (37) and younger age-groups. Thus, it is important to consider developing new equations or cross-validating existing equations on the target population before its use. However, cross-validation of these equations in an independent sample was not possible in this study as we do not have a separate dataset with deuterium dilution measurements. Some authors employ cross-validation by splitting their sample in two but we feel that this is a crude and inefficient method: it only uses half the data to estimate the equation and then only half to estimate the error variance. In a validation study in Matlab, equations were found to be inaccurate in predicting FFM and FM compared to assessment by the deuterium oxide dilution technique (34). The models we have used in this analysis are linear regression models. The variance of observations about their predicted values and the adjusted *R* ^2^ values we give should give a better idea of how well the data are fitted by the model.

### Selection of sample and calculation of power

The main MINIMat trial was designed to evaluate the effects of nutritional interventions on birthweight and survival of the offspring and maternal hemoglobin as primary outcomes. Calculations of the original sample size were made on the basis of finding a difference in birthweight. As this article reports secondary outcomes (child growth and body composition), we calculated the differences in child growth that we could detect with the given sample size (*n*=2,735) in any supplementation group in our analyses. For this sample size, 80% power, and 95% probability, we could detect a difference in height measurements of 0.2 standard deviation (SD) score between six food and micronutrient supplementation groups or a difference of 0.12 SD score for two food groups. In the follow-up of child body composition at 54 months of age, for this sample size (*n*=2,290 children), with 80% power and 95% probability, we could detect a difference of 0.19 SD score between any two of the six food and micronutrient supplementation groups and a difference of 0.11 SD score between the two food groups.

The differences in sample size in different studies depend on the number of participants available with exposure and outcome measurements. In the methodological study (34), the arbitrary selection of 200 children (102 boys and 98 girls aged 4–10 years) was a convenient subsample.

### Statistical analysis

Baseline and follow-up characteristics, including socioeconomic status indicators, were compared across the intervention groups. All singleton newborns were included in intention-to-treat analysis. Outcome variables were: measurements of birth size (i.e. birthweight, length) and child growth up to 54 months of age and body composition (FM, FFM) at 54 months of follow-up. Means and SDs were calculated for continuous variables while proportions were calculated for categorical variables. Descriptive statistics were stratified by sex and age. Values were expressed as means and SD. Differences between sexes were assessed using independent *t*-tests. Differences between the categorical variables were compared using Chi-square tests. *T*-tests and analysis of variance (ANOVA) with post hoc Bonferroni corrections were used for comparing group differences. A comparison among the different supplementation groups, mean height-for-age z-scores, and occurrence of stunting throughout the 5-year follow-up was made using a general linear modeling of repeated-measurements ANOVA procedure. Underweight, stunting, and wasting were defined as < − 2 *z* score of weight-for-age (WAZ), height-for-age (HAZ), and weight-for-height z-scores (WHZ), respectively. In repeated-measures analysis, stunting in each follow-up visit was the within-subject factor and a food and/or micronutrient supplementation group was the between-subject factor. Anthropometric indices (WHZ, HAZ, WAZ) and body composition (FFM, FM) were used as indicators of child growth.

In the methodological study, to create novel equations for estimating FFM in this population, FFM values derived from deuterium oxide dilution were used as a reference method, and impedance values were obtained from the Tanita system. The equations were generated by linear regression analysis, and the impedance index (height^2^/impedance) was fitted as a primary predictor in the basic model, which was then developed by adding variables age, sex, and weight as further predictors.

Effects of food, micronutrient, and EBF interventions on body composition at 54 months of age were evaluated by an intention-to-treat analysis using linear regression. Most statistical analyses were performed using the SPSS software (version 14, 17.0) (SPSS Inc., Chicago, IL, USA) and PASW statistics 20.0 (IBM Corporation, Somers, NY, USA) while we used Analyse-it (version 2.22) free software for Bland-Altman plot analyses.

### Ethical aspects

Both Ethical and Research Review Committees of icddr,b approved the MINIMat trial and the follow-up of children at 54 months of age. As the Ethical Board in Uppsala is limited to research conducted in Sweden, the Board considered and approved the part of the research protocol conducted in Sweden. Written informed consent was obtained from all participating mothers and also from parents/guardian of each participating child.

## Results

### General characteristics

Women who participated in the MINIMat trial were aged 14–50 years at enrolment, and their parity varied from 0 to 10. At enrolment, 27% of women were malnourished (BMI <18.5 kg/m^2^), 30% of infants were LBW (birthweight <2,500 g), and 8% of children were pre-term (born <37 weeks of gestation). The mean birthweight of 3,267 singletons was 2,694 g and at 54 months of follow-up, the mean weight was 13.8 kg, and the mean height was 99.8 cm, with no significant differences among groups.

### Participation

In total, 3,267 singleton infants with birth anthropometry born to 4,436 women were enrolled into the MINIMat trial. Details of losses before measurement of birth anthropometry and losses from follow-up after birth anthropometry up to 54 months of age were earlier reported (12). Losses to follow-up before birth anthropometry did not differ among the intervention groups (p = 0.676). Of the 3,267 live singleton births, body composition at 54 months of age was measured in 2,290 children, representing 70% of the original trial live births; 2,526 children completed anthropometry measurements at 54 months of age. Losses to follow-up did not differ across the intervention groups.

In total, 3,214 (72.45%) of the 4,436 mothers were randomized to breastfeeding intervention, of which 2,845 had a live birth (1,417 in EBC group, and 1,428 were in UHM group). At 54 months of age, 2,168 children completed anthropometry (1,092 in EBC group and 1,076 in UHM group). Distribution of pregnant women and their children for the EBC and UHM groups and numbers lost to follow-up were earlier reported (33).

### Food and micronutrient supplementations and child growth from birth to 54 months of age

**Fig. 1:**
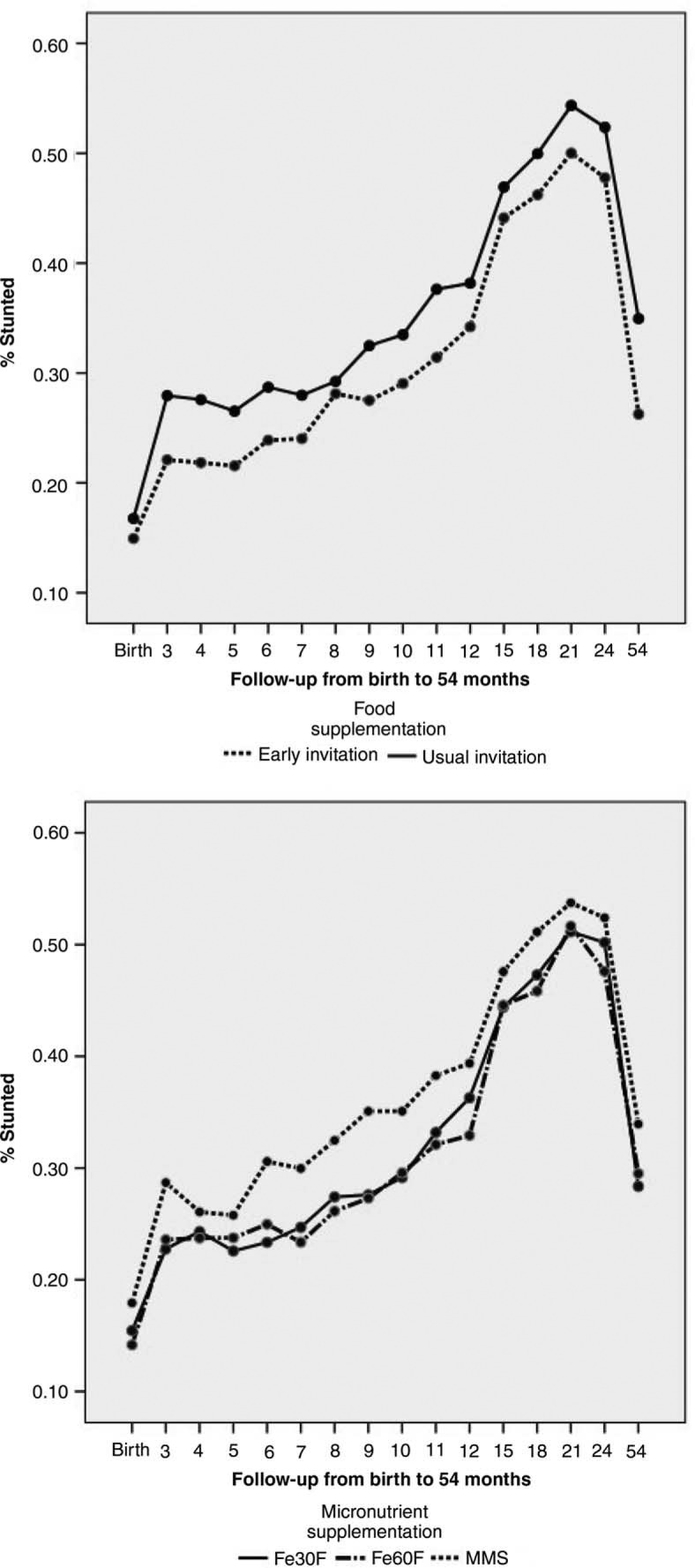
Effects of food and micronutrient supplementations on child linear growth (stunting) from birth to 54 months of age.

No significant differences in characteristics of mothers and households across the different food and micronutrient supplementation intervention groups were observed. A longitudinal analysis of linear growth was performed using repeated-measures analysis (12). There was no interaction between food and micronutrient supplementation on linear growth. Early invitation to prenatal food supplementation to pregnant mothers resulted in a significantly reduced proportion of stunting (Fig. 1) (mean difference–4.5 percentage units, 95% CI 1.2–7.8, p = 0.01). In contrast, MMS resulted in significantly more stunting (Fig. 1) compared to Fe60F (mean difference–4.8 percentage units, 95% CI 0.8–8.9, p = 0.02) (12). The effect of early vs. usual invitation to food supplementation on frequency of stunting was significantly shown for boys but not for girls (Table 1). An increased proportion of stunting in the MMS group was also more expressed among boys (12). Table 2 shows the effects of food and micronutrient supplementation on child growth from birth to 54 months of age, stratified by maternal BMI groups, using BMI of 18.5 as the cut-off level, as original food supplementation by the Government of Bangladesh used this cut-off level for selecting malnourished women for the food-supplementation program. Stunting was less frequent (*p*=0.05) among mothers with higher BMI (>18.5) in the early-invitation food group (compared to usual invitation) while it was not significant among mothers in the lower half of the BMI distribution (*p*=0.10). This trend was also observed when maternal BMI used the cut-off level as median 19.7 (12). Furthermore, the negative effect on linear growth by MMS was also observed in the strata with higher maternal BMI but not significantly different in the group where the mothers had lower BMI (Table 2).

**Table 1:** Effects of prenatal food and micronutrient supplementations on linear growth (16 anthropometric assessments) from birth to 54 months of age, stratified for sex of the child (general lineal models, repeated-measure analyses)

		Boys					Girls				
			
Randomized intervention		No.	HAZ mean (95% CI)	*p*^a^	Stunting, mean% (95% CI)	*p* a	No.	HAZ mean (95% CI)	*p* a	Stunting, mean% (95% CI)	*p* a
Food supplementation	Early invitation (E)	439	−1.55 (−1.64, −1.46)	0.20	31.9 (28.6, 35.2)	0.01	400	−1.50 (−1.58, −1.42)	0.21	29.8 (26.5, 33.0)	0.31
	Usual invitation (U)	405	−1.63 (−1.72, −1.54)		38.4 (34.9,41.8)		390	−1.57 (−1.66, −1.49)		32.1 (28.9, 35.4)	
Micronutrient supplementation	Fe30F	275	−1.50 (−1.61, −1.39)	0.06	32.5 (28.3, 36.7)	0.01	243	−1.52 (−1.63, −1.42)	0.80	31.1 (26.9, 35.2)	0.82
	Fe60F	289	−1.58 (−1.69, −1.47)		32.5 (28.4, 36.6)		277	−1.52 (−1.62, −1.42)		30.0 (26.1, 33.9)	
	MMS	280	−1.69 (−1.80, −1.58)		40.3 (36.2, 44.5)		270	−1.56 (−1.66, −1.46)		31.8 (27.8, 35.7)	
Interaction Food*micronutrients	E-Fe30F	142	−1.44 (−1.60, −1.29)	0.70	28.8 (23.0, 34.7)	0.84	116	−1.51 (−1.66, −1.35)	0.58	31.2 (25.2. 37.2)	0.52
	E-Fe60F	149	−1.57 (−1.72, −1.42)		30.3 (24.6, 36.0)		142	−1.44 (−1.56, −1.30)		27.0 (21.5, 32.4)	
	E-MMS	148	−1.63 (−1.78, −1.48)		36.5 (30.8, 42.2)		142	−1.55 (−1.69, −1.41)		31.1 (25.7, 36.6)	
	U-Fe30F	133	−1.56 (−1.72, −1.40)		36.2 (30.2, 42.2)		127	−1.54 (−1.69, −1.40)		30.9 (25.1, 36.7)	
	U-Fe60F	140	−1.58 (−1.73, −1.43)		34.8 (28.9, 40.6)		135	−1.60 (−1.74, −1.46)		33.1 (27.5, 38.6)	
	U-MMS	132	−1.76 (−1.91, −1.60)		44.1 (38.1, 50.2)		128	−1.58 (−1.72, −1.43)		32.5 (26.7, 38.2)	

CI = confidence interval; HAZ = height-for-age *z* score; Fe30F = 30-mg iron and 400-µg folic acid; Fe60F = 60-mg iron and 400-µg folic acid; MMS = multiple micronutrient supplementation, 15 micronutrients including 30-mg iron and 400-µg folic acid.

a Test of between-subject effects.

**Table 2:** Effects of prenatal food and micronutrient supplementations on linear growth (16 anthropometric assessments) from birth to 54 months of age, stratified for level of maternal BMI (general lineal models, repeated-measure analyses)

		Mother’s BMI <18.5 in early pregnancy					Mother’s BMI ≥18.5 in early pregnancy				
			
Randomized intervention		No.	HAZ mean (95% CI)	*p* a	Stunting, mean% (95% CI)	*p* a	No.	HAZ mean (95% CI)	*p* a	Stunting, mean% (95% CI)	*p* a
Food supplementation	Early invitation (E)	229	−1.67 (−1.79, −1.55)	0.18	36.1 (31.4–40.7)	0.10	606	−1.47 (−1.54, −1.40)	0.26	28.9 (26.2–31.6)	0.05
	Usual invitation (U)	220	−1.79 (−1.90, −1.67)		41.7 (37.0–46.4)		574	−1.53 (−1.60, −1.46)		32.7 (30.0–35.5)	
Micronutrient supplementation	Fe30F	146	−1.74 (−1.88, −1.59)	0.99	38.9 (33.1–44.7)	0.81	370	−1.43 (−1.52, −1.34)	0.02	29.0 (25.6–32.4)	0.02
	Fe60F	157	−1.72 (−1.86, −1.58)		37.6 (32.0–43.1)		407	−1.48 (−1.56, −1.39)		28.8 (25.5–32.1)	
	MMS	146	−1.72 (−1.87, −1.58)		40.2 (34.4–46.0)		403	−1.59 (−1.68, −1.51)		34.6 (31.3–37.9)	
Interaction Food*Micronutrients	E-Fe30F	68	−1.71 (−1.92, −1.49)	0.69	36.4 (27.9–44.9)	0.93	188	−1.39 (−1.52, −1.27)	0.94	27.7 (22.9–32.5)	0.86
	E-Fe60F	81	−1.69 (−1.89, −1.49)		35.3 (27.6–43.1)		209	−1.44 (−1.56, −1.32)		26.2 (21.7–30.8)	
	E-MMS	80	−1.61 (−1.81, −1.42)		36.5 (28.7–44.3)		209	−1.58 (−1.70, −1.46)		32.7 (28.2–37.3)	
	U-Fe30F	78	−1.77 (−1.97, −1.57)		41.3 (33.4–49.3)		182	−1.46 (−1.59, −1.33)		30.3 (25.4–35.2)	
	U-Fe60F	76	−1.75 (−1.96, −1.55)		39.8 (31.8–47.8)		198	−1.52 (−1.64, −1.39)		31.3 (26.7–36.0)	
	U-MMS	66	−1.83 (−2.01, −1.62)		43.9 (35.3–52.5)		194	−1.61 (−1.73, −1.49)		36.5 (31.8–41.2)	

CI =confidence interval; HAZ = height-for-age *z* score; Fe30F = 30-mg iron and 40-µg folic acid; Fe60F = 60-mg iron and 400-µg folic acid; MMS = multiple micronutrients, 15 micronutrients including 30-mg iron and 400-µg folic acid.

a Test of between-subject effects.

### Development of leg-to-leg bioelectrical impedance equation

Characteristics of body composition from deuterium oxide dilution technique were compared with the Tanita scales inbuilt equations in a subsample (*n*=66) of children aged 7–10 years. For boys, TBW (*p*=0.001) was underestimated by the Tanita system whereas there was no difference in TBW measured by both deuterium dilution and the Tanita system for girls (34). However, FM was underestimated (*p*=0.023) and FFM was overestimated (*p*= < 0.001) by the Tanita system (34). Linear regression analysis was used for developing new equations for estimation of FFM. The best-fit equation to predict FFM from linear regression modeling was achieved by adding variables, such as weight, sex, and age to the basic model, bringing adjusted *R*^2^ to 89% (standard error = 0.90, *p*<0.001) that explained 89% of variance in FFM estimated by a deuterium oxide dilution method (34).

### Food and micronutrient supplementation and body composition of children at 54 months of age

We further assessed the effects of food and micronutrient supplementation on child nutritional status and body composition at 54 months of age by an intention-to-treat analysis using unadjusted and adjusted linear regression models (32). No differences were observed in anthropometry or body composition between children in early and usual start of food supplementation groups. There was also no interaction between food and micronutrient supplementation on body composition of children at 54 months of age (FFM, *p*=0.649; FM *p*=0.695) (32).

### Exclusive breastfeeding intervention and child growth and body composition at 54 months of age

Effects of EBF counseling on child growth trajectory from birth to 54 months of age and body composition at 54 months of follow-up were also assessed by intention-to-treat analysis. Breastfeeding counseling intervention was effective in promoting EBF among women in the intervention arm; the mean duration of EBF in the EBC group was 111 days compared to 76 days in the UHM group (mean difference: 35.0, 95% CI 30.6–39.5, *p*<0.001) (33). There was no effect of EBF counseling on child growth trajectories and body composition at 54 months of age (33). There was also no interaction between breastfeeding counseling and food supplementation at 54 months of age. Table 3 shows the mean anthropometry and body-composition measurements at 54 months of age.

**Table 3:** Anthropometry and body composition of children with breastfeeding counseling and usual health message groups in the MINIMat trial at 54 months of age

Characteristics	Exclusive breastfeeding counseling group	Usual health message group
Nutritional status	*n*=1,092	*n*=1,076
Weight (kg)	13.70±1.65	13.73±1.61
Height (cm)	99.57±4.22	99.65±4.25
BMI (kg/m^2^)	13.78±.99	13.80±.93
MUAC (cm)	15.21±1.11	15.23±1.07
Head circum (cm)	47.92±1.50	47.96±1.45
WHZ	−1.31±.86	−1.29±.80
HAZ	−1.57±.92	−1.56±.92
WAZ	−1.81±.87	−1.79±.85
BMI-for-age *z* score	−1.20±.83	−1.18±.77
Wasted (No.,%)	218/1,081 (20.2)	195/1,067 (18.3)
Stunted (No.,%)	347/1,081 (32.1)	340/1,067 (31.9)
Underweight (No., %)	451/1,081 (41.7)	428/1,068 (40.1)
Skinfold (mm)		
Biceps	4.91±1.04	4.89±1.04
Triceps	7.25±1.49	7.30±1.46
Subscapular	5.18±1.10	5.20±1.01
Suprailiac	4.99±1.49	5.03±1.41
Body composition	*n*=997	*n*=973
FFM (kg)^a^	12.10±1.24	12.12±1,22
FM (kg)^a^	1.73±0.59	1.73±0.58
Body fat (%)^a^	12.31±3.28	12.32±3.19
AFA (cm^2^)^b^	5.13±1.28	5.16±1.23
AMA (cm^2^)^b^	18.10±2.65	18.12±2.55

Values are given as mean±SD or No./No. (percentages) where indicated; AFA = arm fat area; AMA = arm muscle area; BF= body fat; BMI = body mass index; FFM = fat-free mass; FM = fat mass; MUAC = mid-upper arm circumference; SD = standard deviation.

a FFM and FM were derived from BIA (Tanita TBF-300MA; Tanita Corporation, Tokyo, Japan) using PoP-specific equations (34).

b Both AFA and AMA were derived from triceps skinfold thickness and MUAC measurements (38).

## Discussion

In this article, we have shown that prenatal nutrition interventions (both positively and negatively) influenced both infant and child growth. Children born to mothers who received early invitation (around 9 weeks of gestation) to food supplementation did not have any differential birthweight and birthlength compared to mothers who started the supplementation with usual program timing (around 20 weeks) but were less likely to become stunted during infancy and childhood. In contrast, children born to mothers who received multiple micronutrients compared to the standard iron–folate program had significantly more stunting during the first 5 years of life, and these effects were primarily observed in boys. The effects of prenatal food and micronutrient interventions on infancy and childhood linear growth suggest fetal programming effects in early life. Neither the nutrition interventions during pregnancy nor EBF counseling intervention had any effect on body composition of children at 54 months of age.

### Effects of prenatal interventions on child growth

Early invitation to food supplementation had a significant effect on linear growth in infancy and childhood. A study in Indonesia reported that a high-energy supplementation during pregnancy reduced the prevalence of childhood stunting throughout the first 5 years compared to a low-energy supplementation (39). The findings of our study suggest that early invitation to food supplementation during pregnancy prevents stunting. Results of an analysis of interventions to prevent stunting in 36 countries showed that nutrition education and counseling on complementary feeding and other supportive strategies could reduce stunting by 19.8% at 12 months of age, 17.2% at 24 months, and 15.0% at 36 months (15). In our study, the level of stunting was reduced by 13% from birth to 54 months of age (difference: 35.3–30.8 = 4.5 percentage units, i.e. a reduction of 13%) when invitation to food supplementation was done early during pregnancy compared to the usual practice. In conclusion, this early prenatal food supplementation reduced the level of stunting in infancy and childhood, with an effect size similar to complementary feeding interventions.

A recent systematic review of prenatal MMS trials reported a mean improvement on birthweight of 52.6 g (40). However, limited information on effects of prenatal MMS on infant and child growth is available. A recent randomized trial in Burkina Faso found that prenatal MMS reduced the stunting rate during infancy by 27% but this effect was no longer observed at 30 months of age (41). One nonrandomized, non-blinded trial of prenatal MMS supplementation in Vietnam found an association with the lower prevalence of stunting in the offspring (42). Combined micronutrient supplementations, mainly iron and zinc, have been evaluated in infants and young children. Results of an Indonesian study among infants suggest that single supplementation with zinc or iron improves infant growth but combined supplementation with iron and zinc had no significant effect on child growth or development (43). Animal experiments have shown that prenatal combined iron and zinc supplementation affects bodyweight and iron status in the offspring (44). Other programming effects on the endocrine system may also have long-term effects on child growth (45, 46). As the micronutrient supplementation was double-blinded, it is unlikely that postnatal care, for example, feeding practices, plays a role in the MMS effect on stunting.

### Effects of prenatal food and micronutrient supplementation on body composition of children

To the best the authors’ knowledge, no study has examined the effects of prenatal food supplementation and multiple micronutrients on body composition of children. We have shown that neither an early invitation to prenatal food supplementation (vs. usual timing) nor MMS (vs. iron–folate supplements) nor any combination of the two resulted in any differences in body composition at 54 months of age, despite the effects on infant and child linear growth.

It is unlikely to have biased the results presented in this study. Anthropometric and body-composition assessments were conducted by only two teams, and each observer performed the same measurement. The pregnant mothers received almost 4 months of daily micronutrient supplements. Allocation to food and micronutrient supplementation was performed according to the protocol, and masking was maintained for the micronutrient supplementation groups until the completion of analysis of the primary outcomes. However, our study has limitations. The study’s children were relatively young and lean at follow-up, and the method used for assessing body composition was BIA, although multicomponent models are now considered sufficiently accurate to measure body composition (distinguishing fat and FFM) (47).

Human (48) and animal studies (49) have shown that there are critical periods in the mother’s dietary intake during pregnancy that can influence future health without altering the size at birth. A study in Peru found that addition of zinc to prenatal folic acid + iron supplementation neither had any effect on birth anthropometric measures nor on body composition of the newborn (50). However, maternal zinc supplementation in this population was associated with better growth of the offspring beginning at month 4 and continuing through to month 12 (51). A recent randomized antenatal micronutrient trial in Nepal (52) found that maternal supplementation with folic acid + iron + zinc resulted in an increased mean height and a reduction in the mean triceps skinfold thickness, subscapular skinfold thickness, and arm fat area (AFA); however, no significant differences among the groups were found in the mean weight or BMI-for-age *z*-scores, waist circumference or arm muscle area (AMA) at 6–8 years of age. Prenatal dietary supplementation to rural Gambian women did not affect body composition of the offspring at 11–17 years of age (53). Thus, our study confirms the findings of other prenatal food or micronutrient supplementation trials that, despite the effects on child growth and other health outcomes, there is weak evidence of an effect on body composition in childhood by such prenatal interventions.

### Timing in pregnancy

Timing of prenatal nutrition interventions may influence the size and scope of responses among offspring, depending, however, on timing of critical periods in pregnancy (49). This has been observed in both animal experimental studies (54) and human observational studies (55). In our study (12), we observed differential effects of timing of prenatal nutrition interventions. An early initiation of food supplements resulted in less-stunted children from birth to 54 months of follow-up compared to the usual initiation of food supplements during pregnancy. Another human study in Guatemala has shown that gain in maternal weight during mid-pregnancy was associated with birthweight, birthlength, and head circumference of the infant, while weight gain in late pregnancy was associated with birthweight, suggesting the importance of timing of nutritional influences in fetal life (56).

### Exclusive breastfeeding intervention

EBF counseling intervention resulted in a substantial increase in the duration of EBF, although EBF counseling intervention compared to the UHM group was not associated with child linear growth and body composition at 54 months of age.

The Promotion of Breastfeeding Intervention Trial (PROBIT), a cluster-randomized trial of a breastfeeding-promotion intervention in the Republic of Belarus, found no significant effect on height, BMI, waist or hip circumference, triceps or subscapular skinfold thickness at 6.5 years of age (57). Although long-term effects of breastfeeding are more difficult to assess, systematic reviews of observational studies found that breastfeeding practices, particularly EBF, are associated with lower blood cholesterol (58) and may be protective against the development of obesity (59). There are also studies that could not demonstrate any association between breastfeeding and anthropometric or body-composition indices (29, 30). In the MINIMat trial, EBF counseling intervention was nested into a prenatal food and MMS, and an analysis of interaction showed that a combination of MMS and EBF counseling had little negative effect on linear growth and higher frequency of stunting at 0–54 months of age. This may point to potential later negative effects by prenatal MMS (44) and the quality and quantity of complementary feeding and its consequences for growth and health outcomes (15).

### Sex differences

Sex differences in fetal and infant growth are well recognized (60, 61). However, the underlying mechanisms are poorly understood. In our study, we found less-stunted children in the early food group (compared to usual group) and more-stunted children in the MMS group (compared to Fe60F). This was evident only in boys (12). The reported difference across sex groups has a biological background (62) but environmental factors or differential treatment may also play a role (boys are often given preference in food allocation in the family in Bangladesh) (63). These observations suggest that male fetuses may be more susceptible to negative influences and more responsive to positive influences. There is also other evidence of sex-specific vulnerability in early fetal growth (64). There is also a sex differential in the effect by prenatal MMS on birthweight, with greater sensitivity in females (21). It is also known that male fetuses demonstrate more rapid growth that makes them more vulnerable in utero (65, 66). Male fetuses are also more sensitive to the nutritional status of women during pregnancy and are, therefore, more susceptible to nutritional constraints whereas girls appear to be more affected by nutritional status over a lifetime (67). The results of our study further support the notion that boys are more vulnerable on maternal nutrition in utero and benefit more from the early start of food supplements than girls.

## Conclusions

Based on the findings of the study, it can be concluded that early invitation to food supplementation compared to usual timing of food supplementation during pregnancy reduces the occurrence of stunting at 0–54 months of age among boys but not among girls. Prenatal MMS, in contrast, increases the proportion of stunting in boys. The effects of these prenatal nutrition interventions on postnatal growth suggest programming effects in early fetal life. Neither the early invitation to food supplementation nor the MMS nor the EBF counseling had an effect on body composition of children at 54 months of age.

In the national program, only pregnant women with BMI of <18.5 kg/m^2^ are invited to receive food supplements. In our study, we found that offspring of women with BMI of >18.5 kg/m^2^ benefitted from early food supplementation.

Previous research from this cohort observed that early food supplementation in combination with MMS substantially decreased infant and under-five mortality (17). Early invitation to food supplementation had also favorable effects on lipid profiles compared to usual food supplementation in early childhood (68). Putting these results together, it clearly shows the beneficial effects of an early invitation to food supplementation and that addition of MMS may reduce the rate of mortality but increase the tendency of stunting of the infant and child. The use of MMS resulted in more stunting (compared to iron–folate), and levels of both insulin and insulin-like growth factors were indicative of slower growth (68). The MINIMat interventions generated important positive results for child health but also had some negative effects. Thus, different trial outcomes point to the necessity of considering not only one but several health outcomes when judging the public-health consequences of an intervention.
